# Internet-Delivered Cognitive-Behavioral Therapy for Social Anxiety Disorder in Romania: A Randomized Controlled Trial

**DOI:** 10.1371/journal.pone.0123997

**Published:** 2015-05-04

**Authors:** Bogdan Tudor Tulbure, Aurora Szentagotai, Oana David, Simona Ștefan, Kristoffer N. T. Månsson, Daniel David, Gerhard Andersson

**Affiliations:** 1 Department of Psychology, West University of Timișoara, Bd. V. Pârvan No. 4, 300223, Timișoara, Romania; 2 Department of Clinical Psychology and Psychotherapy, Babeş-Bolyai University, No. 37 Republici Street, 400015, Cluj-Napoca, Romania; 3 Department of Behavioral Sciences and Learning, Linköping University, SE-581 83, Linköping, Sweden; 4 Department of Clinical Neuroscience, Karolinska Institutet, Stockholm, Sweden, Karolinska University Hospital Huddinge, SE-141 86, Stockholm, Sweden; UNC Chapel Hill, UNITED STATES

## Abstract

**Background and Aims:**

Internet-based cognitive-behavioral therapy (iCBT) for social anxiety disorder has been found effective, as attested by independently conducted randomized controlled trials in four languages. The study aim is to test the efficacy of an iCBT program in a culture where it was not tested before (i.e. Romania).

**Methods:**

Participants (*n* = 76) were recruited, screened and randomized to either a nine-week guided iCBT or a wait-list control group in April and May 2012. Self-report measures were collected before (April 2012) and after the intervention (July 2012), as well as six months later (January 2013). Although social anxiety was assessed with multiple measures, the Liebowitz Social Anxiety Scale - Self Report version (LSAS-SR) and Social Phobia Inventory (SPIN) were used as the primary outcome measures.

**Results:**

A significant difference with a large between-group effect size in favor of iCBT was found (Cohen´s *d* = 1.19 for LSAS-SR and *d* = 1.27 for SPIN). Recovery rates show that 36.8% (*n* = 14) in the treatment group score below the SPIN clinical cut-off compared to only 2.6% (*n* = 1) in the wait-list control group. Post-intervention clinical interviews also revealed that 34.2% (*n* = 13) of the treatment group was completely recovered (full remission) while additionally 36.8% (*n* = 14) retained some social anxiety symptoms (partial remission). However, an important study limitation is that post-intervention interviewers were not blinded to the study conditions. The program also effectively reduced depression and dysfunctional thinking (between-group Cohen´s *d* = 0.84 for depression and *d* = 0.63 for dysfunctional thinking). Moreover, the iCBT intervention appears to have a long-term impact for participants’ functioning, as the treatment gains were maintained six months later.

**Conclusions:**

Internet-delivered interventions display a high potential to quickly and widely disseminate effective evidence-based programs around the world. This study provides support for guided iCBT as a promising treatment approach in Romania.

**Trial Registration:**

ClinicalTrials.gov NCT01557894

## Introduction

Social anxiety disorder (SAD), also referred to as social phobia [1], is defined by the DSM-IV as a persistent fear of one or more social or performance situations in which the person is exposed to evaluation or scrutiny by others. The individual fears that he/she will appear anxious or will act in a manner that will be embarrassing or humiliating [2]. Frequently feared situations include public speaking, talking to strangers or people in authority, and attending public events[3]. DSM-IV criteria for SAD were used throughout the study because it was implemented before the release of DSM-V.

SAD varies in severity and a distinction is commonly made between limited/non-generalized (e.g., public speaking) and generalized SAD [4]. The disorder is highly debilitating, with the majority of socially anxious persons reporting numerous problems in individual and social adjustment [5]. SAD is associated with impairment in academic and professional functioning, as well as in romantic and family relationships [5]. People suffering from the disorder report lower quality of life, are less likely to be married, more likely to divorce, are less educated, and of lower socioeconomic status than people who do not have social anxiety [6]. Moreover, SAD often co-occurs with other psychiatric conditions such as mood disorders and substance use disorders, and is significantly associated with suicidal ideation [5, 7, 8]. In Western cultures, the lifetime prevalence of SAD ranges between 7–12% of the population [9], with low rates of spontaneous remission [10], and prevalence appears to be increasing [11].

There are a range of psychological and pharmacological treatments for SAD [12].The most established psychological treatment for SAD is cognitive-behavioral therapy (CBT), which has proven effective both in individually-administered and group-administered formats[13]. Evidence indicates that individually administered CBT is equally effective [14] or more effective [15]than group CBT and superior to standard psychiatric treatment with medication and emotional support [16].

Cognitive models of SAD emphasize the importance of dysfunctional beliefs in generating the disorder, by transforming innocuous social cues into threats [17, 18]. Thus CBT interventions are mainly focused on altering these beliefs using both cognitive and behavioral strategies such as cognitive restructuring, behavioral experiments, exposure, applied relaxation, and social skills training[19].Previous studies investigated mostly the effect of CBT on cognitive distortions (i.e., description and inferences) (e.g., “Everyone will make fun of me”) and largely ignored the impact on appraisal/evaluative beliefs (i.e., rational and irrational beliefs) (e.g., “They should not make fun of me and if they do this is awful and I am a fool”). In his rational-emotive behavior therapy Ellis[20]argued that irrational beliefs represent core cognitive vulnerability factors for various emotional problems. Therefore, it would be helpful to know whether a CBT intervention could alter both cognitive distortions and evaluative beliefs at the same time.

Despite the availability of effective interventions, only a minority of individuals suffering from SAD seek and receive appropriate treatment [7, 21]. Many people live with their disorder for years before turning to mental health services, and treatment is often sought only when symptoms become too severe and disruptive to manage, or when secondary problems, such as depression or substance use occur [21, 22].Embarrassment associated with help-seeking and fear of what others might think or say have been found to prevent individuals with SAD from seeking treatment [7]. In addition to these disorder-specific issues, barriers to accessing professional assistance such as the lack of skilled therapists, lack of evidence-based treatments, long waiting lists, and costs further prevent accessing appropriate treatment. Therefore, increasing the international availability of evidence-based interventions for people suffering from SAD represents an important challenge [23].

In an attempt to increase the accessibility of adequate, cost-effective treatments, researchers have focused on developing internet-based cognitive-behavioral therapy interventions (iCBT). In most studies, a guided self-help approach has been used, where a web-based presented program is combined with minimal, but regular therapist contact by e-mail or phone [24]. Aside from the advantage of widespread and continuously increasing internet access, the internet version of CBT can be an attractive option for people with SAD, who normally fear and avoid social interactions.

The first iCBT trials for SAD were conducted by Andersson and colleagues [25]using a protocol consisting of nine online modules and two therapist-led exposure sessions. Results indicated large effect sizes that were maintained at 1-year follow-up. Internet-based CBT protocols have since been developed and tested by research groups in Australia, Spain, Sweden, and Switzerland, with results supporting the short- and long-term clinical benefits and financial advantages of these interventions[26]. A recent meta-analysis [27]reported large effects of iCBT on social anxiety symptoms (Cohen’s *d* = .86) and moderate effects on quality of life (*d* = .53), and comorbid anxiety and depression (*d* = .43). Considering these encouraging results, it is important that iCBT for SAD are further examined in randomized clinical trials, with major theoretical and practical objectives.

The first study objective was to investigate the efficacy of any CBT program in a culture where it has not been tested before. Our intervention—called *Internet Social Phobia* (iSOFIE)-was originally written in Swedish[28], being subsequently translated and adapted for use with Romanian SAD clients. Consistent with the principles of parsimony and pragmatism the iSOFIE intervention was reduced, while retaining the main original treatment components. Compared to the previously tested Swedish version, the iSOFIE intervention is shorter in that the amount of information in each module was reduced by half, but the number of modules was unchanged. Such an approach has the potential to effectively treat social anxiety while significantly decreasing the time needed for reading the treatment manual. We assumed that a shorter treatment could reduce the core impairing symptoms in a more parsimonious manner.

Consistent with the evidence-based approach that encourages the effort to test the same program in various contexts and cultures, we investigated the efficacy of an intervention program in a different culture, filling this gap in the literature. Besides enlarging the empirical support of an existing psychological service (an important theoretical contribution), the international availability of an empirically-supported intervention has the potential to alleviate the suffering of many socially anxious individuals in Romania (an important practical contribution).

The second study objective was to look at modifications in appraisal/evaluative beliefs following this brief iCBT intervention. To date, only one study compared the rational-emotive behavior therapy (REBT) with the classical CBT approach and found them equally effective in reducing SAD symptoms[29]. To our knowledge, this is the first study investigating the impact of an iCBT program for SAD on appraisal/evaluative beliefs as secondary outcome measures, making the analysis more nuanced compared to previous studies. Finally, being the first guided iCBT intervention in Romania, an additional objective was to assess participant’s satisfaction with the program.

## Method

The protocol for this trial and supporting CONSORT checklist are available as supporting information (see S1 CONSORT Checklist and S1 Protocol).

### 1.1 Ethics statement

This study was approvedby the Council of Scientific Research Ethics Comission of Babes-Bolyai University, Cluj-Napoca, Romania (Registration No 30273). Written informed consent was obtained from all participants by surface mail.

### 1.2 Study design and sample size

We designed a single-center, parallel group randomized clinical trial (RCT), with equal randomization for the two conditions (i.e., iCBT, and wait-list control). Stratified randomization with diagnostic status as a factor (i.e., clinical and sub-clinical) allowed the wait-list sub-clinical participants to serve as controls for their counterparts allocated to the iCBT condition.

### 1.3 Participants

**Fig 1:**
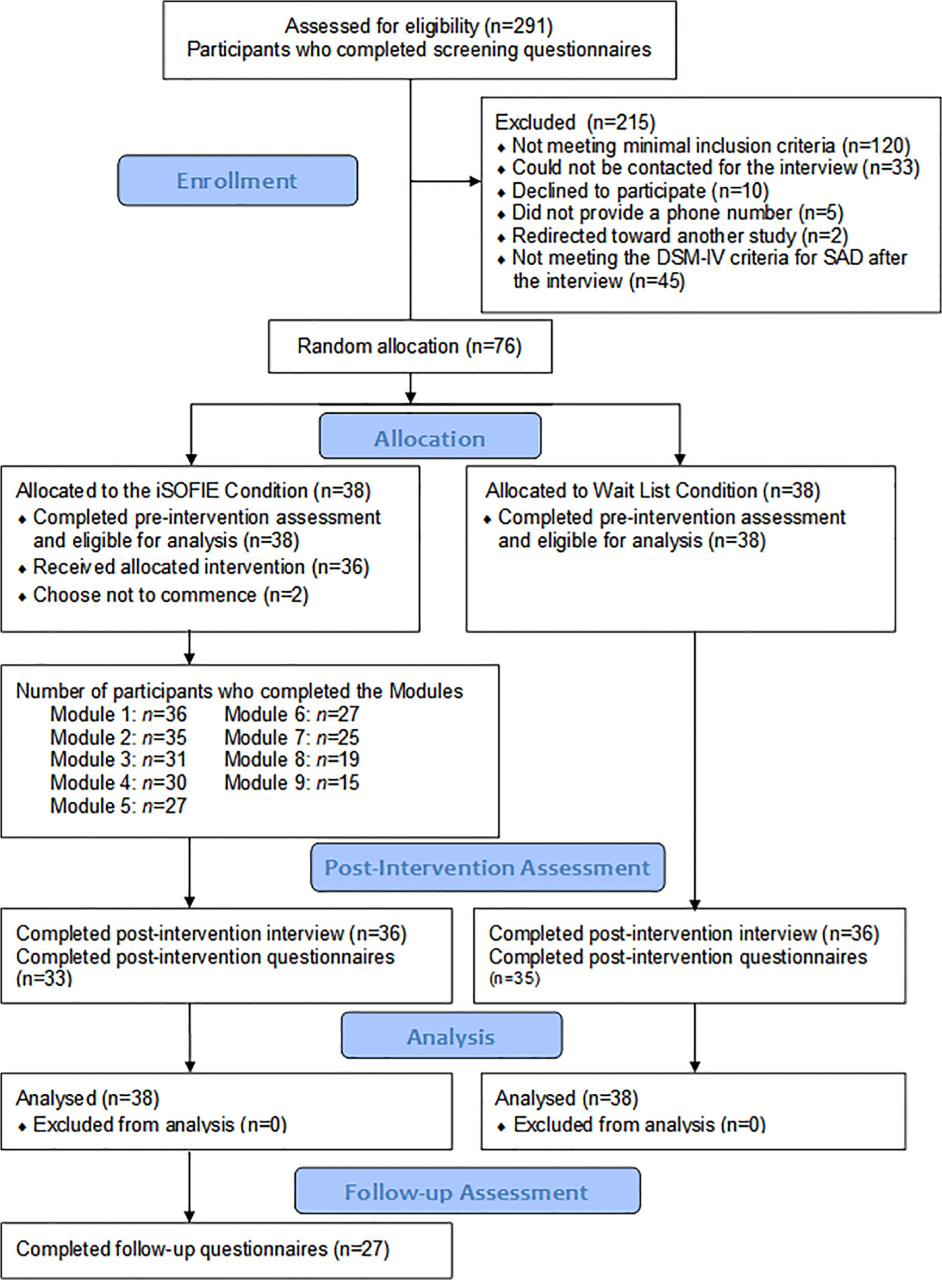
Participants’ recruitment and progress throughout the iSOFIE program.

Volunteer applicants from across the country were screened for the study (*n* = 291). Since the iSOFIE is a disorder-specific program, we only selected participants who could attain the maximum benefits from this intervention (i.e., social anxiety was their major difficulty). After a rigorous check 76participants were recruited for the intervention (see Fig 1). Sixty-six participants (86.84%) meet the DSM-IV criteria for SAD, and ten (13.16%) presented only subclinical symptoms (i.e., they lack one or two criteria to fully meet DSM-IV diagnostic for SAD).Participants’ age ranged from 18 to 54 years, with a mean age of 28.81 (*SD* = 8.04). The demographic characteristics for each of the two groups as well as for the total sample are summarized in Table 1.

**Table 1:** Demographic characteristics of the two conditions.

*Variable*	*iSOFIE*(*N* = 38)		*WLCG*(*N* = 38)		*All participants(N = 76)*		Statistics	*p*
	%	*n*	%	*n*	%	*n*		
Gender								
Male	42.1	16	39.5	15	59.2	45	*χ* ^*2*^ = .05	.81
Female	57.9	22	60.5	23	40.8	31		
Age								
Mean	30.57	-	27.85	-	28.82	-	*F* = .85	.15
(SD)	(7.96)		(7.83)		(8.04)			
Range	18–54		18–53		18–54			
Educational level							*χ* ^*2*^ = 5.54	.23
Master degree	26.3	10	10.5	4	18.4	14		
College degree	47.4	18	47.4	18	47.4	36		
High school degree	23.7	9	36.8	14	30.2	23		
Primary school	2.6	1	5.3	2	3.9	3		
Professional status							*χ* ^*2*^ = 5.39	.25
Full time worker	73.7	28	50.0	19	61.8	47		
Half time worker	2.6	1	5.3	2	3.9	3		
Full time student	18.4	7	36.2	14	27.6	21		
Staying home	5.3	2	5.3	2	5.3	4		
On social support	0.0	0	2.6	1	1.3	1		
Marital status							*χ* ^*2*^ = 1.12	.77
Never married	52.6	20	63.2	24	57.9	44		
In a relationship	21.1	8	13.2	5	17.1	13		
Married	21.1	8	18.4	7	19.7	15		
Divorced	5.3	2	5.3	2	5.3	4		
Previous psychotherapy (within 5 years)	13.2	5	5.3	2	9.2	7	*χ* ^*2*^ = 1.41	.23
Time spent online							*χ* ^*2*^ = 1.65	.30
2 hours / day	18.4	7	21.1	8	19.7	15		
4 hours / day	47.4	18	26.3	10	36.9	28		
> 4 hours / day	34.2	13	52.6	20	43.4	33		

Abbreviations: iSOFIE = the iSOFIE intervention group; WLCG = Wait-List Control Group.

Eligibility criteria for the study included: (a) being over 18 years old, (b) exceeding the cut-off score on *Social Phobia Inventory* (SPIN) (i.e., SPIN total score ≥ 19)[30], *Social Interaction and Anxiety Scale* (SIAS) (i.e., SIAS total score ≥ 24) [31], and *Liebowitz Social Anxiety Scale—Self Report version* (LSAS-SR) (i.e., LSAS-SR total score ≥ 30) [32],(c) fulfilling the DSM-IV criteria for SAD on *Social Phobia Screening Questionnaire* (SPSQ) [33], (d) having SAD as the primary diagnostic on *Structured Clinical Interview for DSM-IV-TR* (SCID) [34], (e) presenting no suicidal ideation (i.e., not exceeding a score of 2 on the suicide item of *Beck Depression Inventory-II* (BDI-II) [35], and not reporting parasuicidal behavior on the *Screening Questionnaire* of the SCID), (f) not currently receiving other forms of psychological treatment for SAD, (g) having access to a computer connected to the internet, (h) if on medication, the dose should be constant for at least 1 month, and participants should agree to keep the dosage unchanged for the whole duration of the study, and (i) having no diagnosis of psychoses or borderline personality disorder on the SCID. The accepted cut-off scores for the SAD measure according to the recommendations found for each instrument in their original article were used. However, because SPIN has the most reliable empirical support for its clinical cut-off, the recovery rates were estimated only for SPIN.

To be included in the study, participants were allowed to have prior history of treatment for SAD, but in the treatment group only five participants reported receiving treatment within the last five years (which speaks of the low rate of SAD treatment in Romania).

### 1.4 The iCBT Intervention

The present intervention was adapted from a manual [28] previously tested in Sweden[25, 36–39]. Relevant information about social anxiety is presented in each of the nine modules (see Table 2), and participants are asked to fill in essay questions, provide thought records, build anxiety hierarchies, describe their exposure exercises, and complete a weekly social anxiety measure (LSAS-SR). The iSOFIE participants were encouraged to contact their online psychologist if difficulties in understanding the text or in implementing the practical applications were encountered. A weekly feedback was offered to each participant and questions were answered within a 48-hour interval. In order to proceed through the intervention, participants had to fill in the exercises and essay questions at the end of each module. The access to the next module were given if participants’ responses prove that they had a correct understanding of the main concepts (i.e., they know the difference between thoughts and feelings, they understand the role of safety behaviors etc.) and they used them in at least one real life context. Participant’s subjective understanding was assessed though the weekly essay questions and homework online assignments. The iSOFIE participants were granted one week for each module, thus the nine-week intervention time-frame. The control group received no active treatment during the nine-week interval, but participants were asked to complete a weekly social anxiety measure (LSAS-SR).

**Table 2:** Brief Description of the Romanian iSOFIE intervention program.

No.	Module title	Module description	Worksheets / Exercises
1.	Introduction	A description of the treatment program (content and structure), and of social anxiety (common symptoms, causes and treatment strategies) are presented in the first module.	■ Select your problem area
			■ Therapy goals
			■ Essay questions
2.	The negative automatic thoughts	The role of negative automatic thoughts and the Clark & Wells cognitive model of social phobia are detailed. Information about assumptions and rules are provided as supplementary reading.	■ The negative automatic thought record
			■ Fill in your vicious circle
			■ Essay questions
3.	Challenging negative automatic thoughts	The main strategies for challenging negative thinking (examining the evidence, all or nothing thinking, taking someone else's perspective, worst case scenario) and a list of cognitive distortions are presented.	■ The dysfunctional thought record (x3)
			■ Essay questions
4.	Behavior Experiments	Common problems that may arise when identifying and challenging negative automatic thoughts and behavioral experiments are introduced as way to test negative thoughts.	■ Behavioral experiments
			■ Essay questions
5.	Exposure	Exposure principles are introduced. Participants are instructed to create an anxiety hierarchy and encouraged to gradually approach the feared situations.	■ Creating an anxiety hierarchy
			■ Exposure 1
			■ Essay questions
6.	Exposure and self-focus attention	Self-focus attention and its role in maintaining social phobia is presented. A number of strategies to reduce self-focus attention and the role of safety behaviors are also described.	■ Exposure 2
			■ Experiments with (and without) safety behaviors
			■ Essay questions
7.	Exposure and getting closer to your fears	Solutions to difficulties that arise in connection with exposure are amply supplied here. Suggestions for exposure situations and strategies to confront the worst fears are also offered.	■ Exposure 3
			■ Confront your worst fears
			■ Essay questions
8.	Social skills	A number of techniques to stimulate participants’ social skills (active listening, communication, assertiveness, saying “no”), are offered and participants are encouraged to use them.	■ Exposure 4
			■ Behavioral experiment (social training)
			■ Essay questions
9.	The maintenance plan	Information about relapse prevention and maintenance of treatments gains are finally offered. The supplementary reading presents information on perfectionism and self-confidence.	■ The maintenance plan
			■ Essay questions
			Total 24 Exercises

### 1.5 Procedure

The trial was briefly presented in various national and local newspapers (April, 2012), where a link to the project website (https://www.iterapi.se/sites/isofie) was provided. Interested participants registered online, read the informed consent, and filled in the screening questionnaires over the internet. Those who fulfilled the minimal inclusion criteria (i.e., high levels of social anxiety, low levels of depression, over 18 years, declared to have no psychosis or personality disorder, and were not currently treated for social anxiety) were contacted for a telephone interview. The interview was conducted within 12 days after the completion of the screening, and was based on the SCID. The purpose of this interview was to assess the applicant’s diagnostic status according to the DSM-IV criteria for SAD, and to briefly check for possible major problems undetected during screening. Sixty-six applicants (out of 121 who were contacted for the interview) meet the DSM-IV criteria for SAD. Ten additional applicants presented subclinical symptoms (i.e., they lack one or two criteria to fully meet DSM-IV diagnostic for SAD). In total, 76 applicants were included in the study as we predicted that they could benefit from this low-intensity psychosocial intervention. The flow of participants throughout the study is diagramed in Fig 1.

Following assessment, the 76 included participants were randomly assigned to either the iSOFIE intervention (*n* = 38) or to the wait-list control condition (*n* = 38) via a computerized randomization procedure (May, 2012; see the randomization procedure). Participants in both conditions received an email with information about the assigned group, and were invited to perform a different set of tasks. The wait-list control participants were asked to fill in a weekly social anxiety measure (LSAS-SR) for the following nine weeks, and were informed that in ten weeks they will benefit from the active treatment. The iSOFIE participants were given access to the first module and were also invited to fill in the weekly social anxiety measure (LSAS-SR). Moreover, each iSOFIE participant was randomly assigned to an online psychologist who assisted him/her throughout the intervention. The psychologists were responsible to monitor participant’s activity (i.e., send reminders when no activity was seen on the platform, provide feedback for the homework assignments, answer participant’s questions etc.). All participants were supported throughout the treatment, and positive encouragements were provided for every noticeable progress.

The post-intervention assessment (July, 2012) consisted of a short telephone interview (i.e., the SCID Social Anxiety Module) and the same online questionnaires as the ones completed during the screening phase. In order to assess the satisfaction with the intervention, the iSOFIE participants were further invited to fill in a treatment satisfaction questionnaire. After the post-intervention week (i.e., week ten), participants were no longer able to communicate on the platform, but they were able to access all the modules for the next six months (until the follow-up assessment conducted in January, 2013). Finally, control group participants started the iCBT intervention one week after the iSOFIE group completed the program.

### 1.6 Measures

#### 1.6.1 Primary outcome measures

The *Liebowitz Social Anxiety Scale—Self-Report version* (LSAS-SR) [32] presents 24 commonly anxiety-provoking situations, and asks participants to rate their fear and avoidance for each situation. The psychometric properties of the LSAS-SR are good to excellent [40] and the scale captures symptom changes in both cognitive-behavioral and psychiatric interventions. As a result, the LSAS-SR was recently rated among the evidence-based measures, and recommended for treatment monitoring and treatment outcome in adults[41] and adolescents[42].

The *Social Phobia Inventory* (SPIN) [30], is a brief (i.e., 17-item) self-report instrument measuring fear in social situations, avoidance of performance/social events, and physiological discomfort in social situations. Each item is rated on a four-point scale, with higher scores corresponding to greater distress. The scale has generally good to excellent psychometric proprieties[30, 43]and was considered an evidence based measure for treatment monitoring and treatment outcome[41].

#### 1.6.2 Secondary outcome measures

Besides these primary outcome measures, social anxiety symptoms were assessed with two additional measures. The *Social Interaction and Anxiety Scale* (SIAS) [31], is a 20-item measure that assesses fears of general social interactions. The scale captures both social scrutiny fears and social interaction fears. For each item, respondents are asked to indicate the degree to which they feel the statement is characteristic or true of them on a five-point scale. The SIAS was found to have sound psychometric properties [31].

The *Social Phobia Screening Questionnaire* (SPSQ)[33], is a diagnostic questionnaire for SAD. The measure presents both dimensional and categorical data, including impairment and duration of reported social anxiety [33]. SPSQ was previously used in various SAD RCTs [25, 37, 38].

#### Depressive symptoms

The severity of depressive symptoms according to DSM-IV was measured with the *Beck Depression Inventory-II* (BDI-II) [35]. Each item consists of four statements indicating increasing symptom severity. Sound psychometric proprieties were reported for BDI-II [35] and it was evaluated among the evidence-based measures for treatment outcome [44].

#### Cognitive outcomes

Cognitive factors related to emotional problems were explored to see whether the iCBT program significantly contributes to their reduction (e.g., automatic negative thoughts, irrational thinking).

Participants’ negative thoughts and related cognitive processes (most of them descriptions/inferences) were measured with the *Automatic Thoughts Questionnaire* (ATQ) [45] and irrational thinking patterns (i.e., all of them evaluative beliefs) were measured with the *Attitude and Belief Scale-II* (ABS-II) [46, 47]. Although ABS-II allow us to compute various scores (e.g., for rational beliefs, for specific irrational beliefs), we focus here only on the total score of irrational beliefs as a first step to investigate possible changes generated by the iCBT.

### 1.7 Randomization

Once registered on the treatment platform, participants received a unique study code. An independent person was asked to randomly assign the codes to one of two conditions (using a 1:1 allocation procedure) using a computer-generated randomization procedure with stratification by diagnostic status (clinical and subclinical) as an additional criterion. The interviewers were blind to which group the participants had been randomized. However, because the difference between the iSOFIE and the control condition could not be masked, online therapists and participants were not blinded regarding the condition assignment.

### 1.8 Statistical Analyses

At baseline, group differences in demographics were analyzed using the *t*-test and the Chi-square test. Because not all participants completed every weekly, post-test or follow-up measure, the missing data (representing 7.4% of all data) were imputed with multiple imputation procedures [48]. No pattern was established for the relatively small amount of missing data. Therefore, multiple imputation to predict the random missing data was used. Changes in both primary and secondary outcome measures were evaluated using Univariate ANCOVA, with pre-intervention scores as covariates, and group as a fixed factor[49]. Effect sizes were calculated using Cohen’s *d* for both within- and between-group comparisons, with the pooled standard deviation of the compared groups as denominator. Paired sample *t*-tests were used to test whether treatment gains were maintained between post-intervention and the six-month follow-up assessment. Finally, 95% confidence intervals (CI) were used to estimate the precision of the effect size for all measures. All analyses were performed in SPSS version 17.0 (SPSS, Inc., Chicago, IL).

### 1.9 Clinical Responder Definition and Significant Change

Recovery rates based on patient’s self-reported social anxiety was estimated by the proportion of patients who scored below the clinical cut-off at the post-intervention assessment. Participants who did not meet the DSM-IV criteria for SAD at the post-intervention SCID interview (i.e. were evaluated as having less than two SAD symptoms) were considered responders. However, participants who did not meet the DSM-IV criteria for SAD but retained some residual symptoms at post-intervention were considered in partial remission. Clinically significant change was calculated on the main outcome measures using a conservative method suggested by Jacobson and Truax [50]. The participant’s change had to be more than two SDs from the group mean symptom level at baseline (i.e., all included participants), as well as the level had to be within two SDs from a non-clinical reference group [30, 51]. LSAS-SR total scores less than 39.53, and SPIN total scores less than 28.52 defined clinically significant change in the present study.

## Results

### 2.1 Pre-intervention Assessment

The two conditions displayed similar demographic characteristics, and no statistically significant group differences were observed in terms of age, gender, educational level, professional and marital status, and previous psychotherapy attendance at baseline (i.e., all *F* and *χ* ^*2*^displaying a *p* between. 15 and. 81). Further details about the demographic characteristics are presented in Table 1. The screening self-report measures used for both groups displayed the following internal consistencies (Cronbach’s Alpha): LSAS-SR = .92; SPIN = .85; SIAS = .80; SPSQ = .82; BDI-II = .80; ABS-II = .84; ATQ = .88.

Although social anxiety symptoms were significantly interfering with their normal life routine, none of the 76 included participants were using psychoactive medication nor were they involved in any kind of psychotherapy when assessed for the present study. This alone highlights the lack of SAD treatments in Romania.

### 2.2 Adherence and attrition

At the end of each module participants could save their answers on the iSOFIE platform. Treatment adherence was estimated by the number of completed worksheets (i.e., ranging between zero and 24). On average, participants completed 2.6 weekly worksheets. Overall, the iSOFIE participants completed 662 worksheets (*mean* = 17.43, *SD* = 7.77), and the number of completed modules is presented in the flow diagram (Fig 1). Post-intervention questionnaires were collected from 68/76 participants (89%), while post-intervention SCID interviews were conducted with 72/76 participants (94%). Finally, six-month follow-up questionnaires were collected from 27/36 participants (75%).

### 2.3 Primary outcome measures

Univariate ANCOVAs on post-intervention LSAS-SR and SPIN, controlling for pre-intervention scores, revealed that the iSOFIE group displayed significantly lower post-intervention scores compared to the control group scores (see Table 3 -first two rows). Separate analyses were conducted only for clinical participants (the 66 participants who meet the SAD diagnostic criteria at pre-intervention interview) to see whether these results are in any way different when compared to the results obtained from all participants. No significant differences could be observed (see Table 3).

**Table 3:** Results of the two groups presented separately for all / for only clinical participants.

*Statistical analyses/number of participants analyzed*	*LSAS-SR*	*SPIN*	*SIAS*	*SPSQ*	*BDI-II*	*ATQ*	*ABS-II*
***Results for the first intervention group (the iCBT group)***							
*Univariate ANCOVA (comparing pre- to post-intervention scores)*							
Clinical and subclinical Ps (*n* = 76)	34.12**	43.30**	44.71**	43.74**	18.11**	9.72**	8.46**
Only clinical Ps (*n* = 66)	31.28**	44.13**	40.82**	39.44**	16.16**	12.04**	5.83**
*Paired sample t-test (comparing post-intervention to six-month follow-up scores)*							
Clinical and subclinical Ps (*n* = 76)	1.39	3.43**	3.27**	2.75*	.10	2.26*	2.59**
Only clinical Ps (*n* = 66)	.89	3.13**	3.00**	2.35*	-.22	2.58*	2.33*
***Results for the second intervention group (the former wait-list control group)***							
*Paired sample t-test (comparing pre- to post-intervention scores)*							
Clinical and subclinical Ps (*n* = 33)	8.39**	9.29**	7.18**	8.93**	5.71**	5.52**	5.84**
Only clinical Ps (*n* = 28)	7.29**	8.89**	6.35**	7.87**	4.29**	5.15**	5.09**

Notes: 1) The values presented in Table 3 represent the Univariate ANCOVAs and the paired sample t-test for the main outcome measures. 2) The wait-list control group (WLCG) received the same psychosocial intervention after the iCBT group ended it (i.e., starting from week ten). The data presented in the bottom of Table 3 summarized the differences between the pre- and post-intervention scores obtained by the second intervention group (i.e. the former WLCG). Abbreviations: LSAS-SR = Liebowitz Social Anxiety Scale–Self-Report version; SPIN = Social Phobia Inventory; SIAS = Social Interaction and Anxiety Scale; SPSQ = Social Phobia Screening Questionnaire; BDI-II = Beck Depression Inventory-II; ATQ = Automatic Thoughts Questionnaire; ABS-II = Attitude and Belief Scale-II. Ps = Participants.

** *p* <. 01

* *p* <. 05

**Fig 2:**
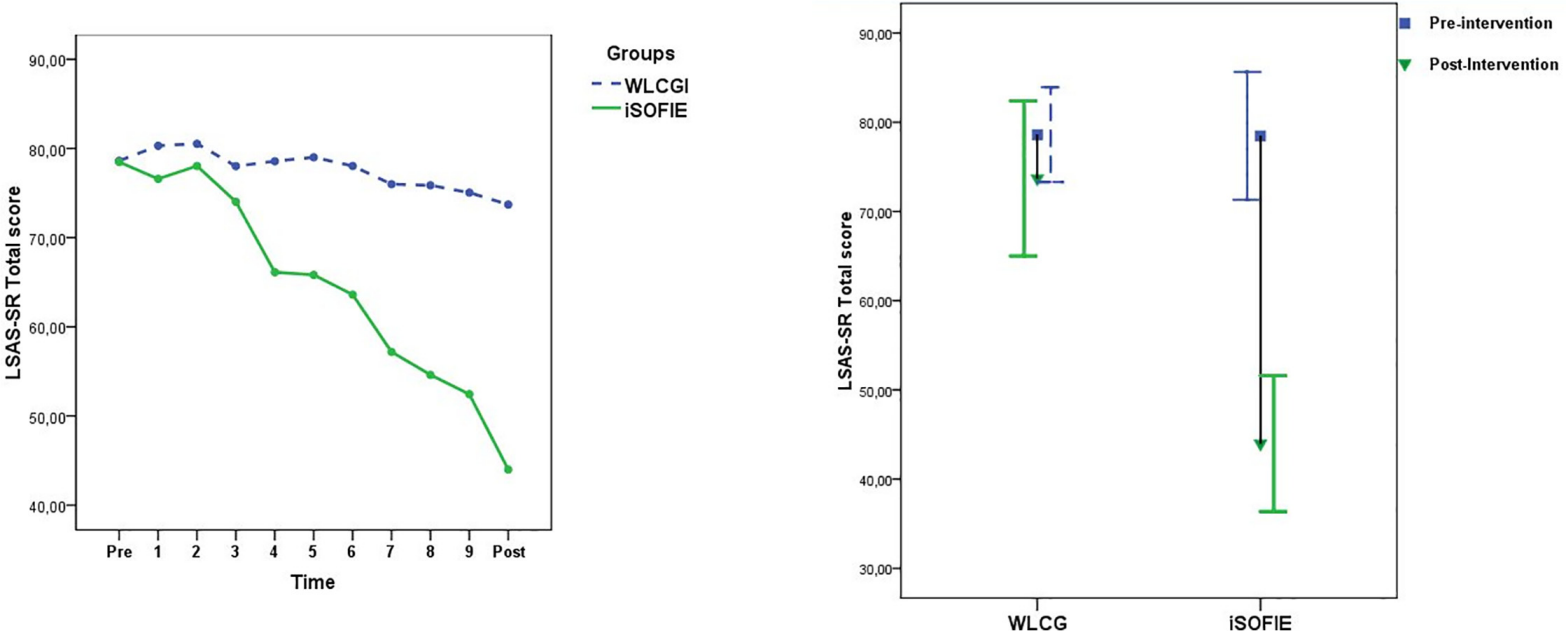
Social anxiety levels for the two groups throughout the iSOFIE program. Abbreviations: iSOFIE = the iSOFIE intervention group; WLCG = Wait List Control Group; Pre = Pre-intervention assessment, Post = Post-intervention assessment.

Large between-group effect sizes (Cohen´s *d*) were observed at post-intervention for all measures (*ES* ranged between 1.19 and 1.36; see Table 4). The social anxiety levels captured by the weekly LSAS-SR for both groups are depicted in Fig 2A. The 95% CI of the iSOFIE and WLCG do not overlap at post-intervention, illustrating that the interval estimates of the two groups are divided by an important gap (see Fig 2B).

**Table 4:** Results of the two groups before and after the iSOFIE program (all participants).

*Group / Measures*	*Pre-intervention assessment Mean (SD)*	*Post-intervention assessment Mean (SD)*	*Follow-up assessment Mean (SD)*	*Pre to Post within-group ES (95% CI)*	*Post-intervention between-group ES (95% CI)*	*Pre to Follow-up within-group ES (95% CI)*
**The first treatment group (the iSOFIE group)**						
*Primary outcome measures*						
LSAS-SR	78.47 (21.78)	43.98 (23.17)	35.77 (25.44)	1.53 (1.01 to 2.03)	1.19 (0.70 to 1.67)	1.84 (1.23 to 2.40)
SPIN	45.15 (9.69)	27.95 (13.04)	19.74 (13.00)	1.50 (0.97 to 1.99)	1.27 (0.76 to 1.75)	2.33 (1.76 to 2.93)
SIAS	50.21 (11.10)	32.94 (12.19)	25.40 (12.98)	1.48 (0.96 to 1.97)	1.35 (0.84 to 1.83)	2.08 (1.45 to 2.66)
SPSQ	31.47 (8.43)	16.47 (8.93)	11.60 (9.65)	1.73 (1.18 to 2.24)	1.36 (0.85 to 1.85)	2.23 (1.56 to 2.83)
*Secondary outcome measures*						
BDI-II	15.15 (7.47)	7.25 (5.95)	6.44 (7.05)	1.17 (0.67 to 1.64)	0.84 (0.35 to 1.29)	1.19 (0.65 to 1.61)
ATQ	32.76 (7.53)	25.24 (8.26)	21.48 (6.85)	0.95 (0.47 to 1.42)	0.69 (0.22 to 1.14)	1.55 (0.96 to 2.10)
ABS-II	120.94 (39.78)	76.62 (34.81)	57.96 (31.67)	1.19 (0.69 to 1.66)	0.63 (0.16 to 1.08)	1.72 (1.12 to 2.27)
**WLCG**						
*Primary outcome measures*						
LSAS-SR	78.60 (16.14)	73.69 (26.46)		0.22 (-0.23 to 0.67)		
SPIN	45.50 (9.45)	43.22 (10.94)		0.22 (-0.23 to 0.67)		
SIAS	52.10 (12.61)	50.66 (14.04)		0.11 (-0.34 to 0.56)		
SPSQ	31.42 (8.49)	30.05 (10.88)		0.14 (-0.31 to 0.59)		
*Secondary outcome measures*						
BDI-II	15.39 (6.99)	12.77 (7.27)		0.37 (-0.09 to 0.82)		
ATQ	33.34 (9.32)	31.57 (10.05)		0.18 (-0.27 to 0.63)		
ABS-II	124.60 (41.50)	102.51 (46.62)		0.50 (0.04 to 0.95)		
**The second treatment group (the former WLCG)**						
*Primary outcome measures*						
LSAS-SR	75.66 (24.44)	40.29 (19.56)		2.58 (-5.25 to 9.58)		
SPIN	44.06 (11.46)	23.82 (8.44)		3.23 (-0.68 to 6.07)		
SIAS	51.45 (14.81)	32.92 (10.87)		2.31 (-2.74 to 6.02)		
SPSQ	30.33 (11.26)	13.22 (6.62)		3.06 (-0.78 to 5.32)		
*Secondary outcome measures*						
BDI-II	13.00 (7.67)	5.60 (5.64)		1.78 (-0.84 to 3.70)		
ATQ	31.54 (10.55)	21.70 (5.22)		2.00 (-1.60 to 3.78)		
ABS-II	104.51(49.30)	59.48 (29.46)		1.83 (-14.99 to 11.88)		

Abbreviations: LSAS-SR = Liebowitz Social Anxiety Scale–Self-Report version; SPIN = Social Phobia Inventory; SIAS = Social Interaction and Anxiety Scale; SPSQ = Social Phobia Screening Questionnaire; BDI-II = Beck Depression Inventory-II; ATQ = Automatic Thoughts Questionnaire; ABS-II = Attitude and Belief Scale-II.ES = Effect size; WLCG = wait list control group. Note: For the first three columns the values represent the self-report mean and (SD = standard deviation), while for the last three columns the values represent the ES and (95% CI = confidence interval).

### 2.4 Secondary outcome measures

Univariate ANCOVAs were also conducted on the secondary outcome measures, controlling for pre-intervention scores. Results showed that the iSOFIE group displayed lower post-intervention scores when compared with the control group on depression, negative automatic thoughts, and irrational thinking scores (see Table 3). Effect sizes ranged from moderate to large (between-group *ES* = 0.63 to 0.84, and within-group *ES* = 0.95 to 1.19; see Table 4).

### 2.5 Six-month Maintenance

Paired-sample *t*-tests comparing post-intervention to six-month follow-up scores revealed significant reductions on most primary and secondary outcome measures: SPIN, SIAS, SPSQ, ABS-II, and ATQ (see Table 3- rows three and four). In the absence of any additional interventions participant’s social anxiety and irrational thinking displayed lower levels after six months. No significant improvements from post-intervention to follow-up were observed for LSAS-SR, and BDI-II (see also Table 3). However, pre- to follow-up within-group effect sizes revealed large effects for all measures (Table 4).

### 2.6 Clinical Significance

As concerning the treatment response, a total of 34.20% (*n* = 13) of the participants in the iSOFIE group were considered symptom free (i.e., total remission) at the post-intervention SCID interview. Moreover, 14 additional participants (36.80%) significantly improved as they did not meet the DSM-IV criteria for SAD but maintained some residual social anxiety symptoms at the end of the treatment (i.e., partial remission). Compared to the WLCG (*n* = 4), a significant proportion of the iSOFIE participants (*n* = 27) were considered responders as they obtained either total or partial remission (i.e., *χ* ^*2*^(1) = 28.82; *p*<.01). Recovery rates show that 14 participants (36.80%) from the iSOFIE group and one participant (2.6%) from the WLCG score below the SPIN clinical cut-off (i.e., below 19 points) at post-intervention assessment. Most participants who scored below the SPIN clinical cut-off were also considered in total remission at the post-intervention SCID interview. Moreover, six-month later, in the absence of any intervention, the recovery rates ascended to 55.60% as 15 participants scored beyond the SPIN clinical cut-off.

We further investigated the impact of the treatment using a clinical significant change criterion. The LSAS-SR outcome measure showed that47.4% (18/38) of the iSOFIE participants (i.e., the first treatment group) reached this criterion, versus 13.2% (5/38) in the WLCG. This difference is statistically significant *χ* ^*2*^(1) = 10.53, *p*<.01.Additionally, the SPIN outcome measure showed that 44.7% (17/38) of the iSOFIE participants scores were clinically and significantly changed, and these results were different from the WLCG (7.9%, 3/38; *χ* ^*2*^(1) = 13.30, *p*<.01).For the second treatment phase (i.e., when the former WLCG participants received the iCBT) 44.7% (17/38) met the clinically significant change criterion for LSAS-SR and 60.5% (23/38) for SPIN.

### 2.7 Treatment Satisfaction

At the end of the program, participants who completed the treatment satisfaction questionnaire rated the quality of the iSOFIE modules as *good* and *very good* (28% and 72% respectively). The overall satisfaction with the program was also high, participants being *satisfied* or very *satisfied* with the intervention (40%, 46% respectively). Only a few responders declared to be *neither satisfied nor dissatisfied* with the program (14%), and none rated it as *unsatisfactory*. While the intervention was generally considered *demanding* or *very demanding* (62%, 10% respectively), all responders viewed the iSOFIE program as *helpful* or *very helpful* (28%, 72% respectively). On average participants spent 6.8 hours per week solving the various iSOFIE tasks (e.g., reading the text, completing the homework, writing to their online psychologists etc.) and most of them declared to have had an overall positive experience. Few complaints were made about the limited time available for each module (three participants), the perceived distant communication with her online therapists (one participant) and a remediable technical difficulty (one participant). Finally, participants were asked to indicate their level of agreement on a ten-point scale, where ten indicate the highest agreement. Participants rated the iSOFIE program approach as logical (9/10), and were willing to recommend it to a friend with similar problems (9/10).

### 2.8 Results for the second treatment group (the former wait-list control group)

After the first group completed the intervention, participants from the wait-list control group were given the chance to receive the same intervention. Paired-sample *t*-tests comparing pre- to post-intervention scores for the second treatment group revealed significant reductions for all measures (see last two rows of Table 3). Within-group effect sizes were large (*ES* = 1.78 to 3.23) supporting once again the efficacy of this intervention (see Table 4).Recovery rates show that 30.06% (*n* = 10) of the second treatment group participants scored below the SPIN clinical cut-off at post-intervention. No follow-up data were collected for this group.

## Discussion

This study was a randomized controlled trial aimed to investigate the efficacy of an internet based cognitive-behavioral intervention compared to a wait-list control group in the treatment of SAD. The Romanian iCBT intervention, named iSOFIE, was based on a Swedish internet-delivered CBT protocol which had been tested in previous studies [25, 39, 52].

Overall, the results suggest that the iSOFIE program is effective not only in terms of treating SAD symptoms, but also in reducing depression and dysfunctional thinking (e.g., both distorted and evaluative cognitions). The intervention had a statistically significant effect on social anxiety symptoms, with large effect sizes for all the social anxiety symptoms measures. This supports previous findings presented in the literature [23, 53]. Furthermore, a 36.8% SPIN recovery rates for the iSOFIE group compared to only 2.6% in the WLCG shows that the differences between the two groups are both statistically and clinically significant.

In terms of the secondary outcomes (i.e., depression, automatic, and irrational thinking), significant changes favoring the iSOFIE group were observed, with moderate to large effect sizes. Since the changes in depression were obtained in the absence of direct focus of the treatment, these effects could show generalized effects of the intervention. Indeed, as depression and anxiety symptoms are often comorbid, they may have common cognitive mechanisms (e.g., inflexible/rigid irrational beliefs) that were addressed in the present study. Moreover, the treatment effects appear to be stable for both primary and secondary outcome measures, as most scores remained unchanged or decreased six months after the program ended. It is possible that, once participants did not meet the primary diagnostic criteria for SAD the secondary/associated depression symptoms were also reduced (i.e., due to the decrease of social anxiety). Similar decreases in secondary outcome measures were found in previous studies[25]. In terms of the treatment satisfaction, participants reported being satisfied with the treatment.

Finally, the original SOFIE interventions conducted in Sweden reveal similar medium to large ES for both primary and secondary outcome measures [25, 37, 38]. In a recent study where the SOFIE program was compared to a WLCG using a big sample (*n* = 204), a large between group ES at post-intervention was obtained on the LSAS-SR (Hedge’s *g* = .75;[36]). Interestingly, the parallel delivery of a similar intervention via a smart phone application in Sweden (mSOFIE) also yielded medium to large within-group ES for social anxiety measures (Cohen’s *d* range between. 71 and. 99;[52]). Considering these preliminary results, it appears that condensing treatment manuals (i.e., presenting shorter versions either online or via smart phones) display the potential to effectively reduce social anxiety and related symptoms in a parsimonious manner.

### 3.1 Study limitations

This study presents a number of limitations. First, an active control condition matched for clients’ expectations, time, and therapist involvement was not included. Therefore the obtained results could be due to non-specific factors associated with any active treatment and the reported results should be interpreted with caution (i.e., due to the non-existent match in terms of experimental design). Although we could not demonstrate that our iCBT program impacts participant’s life beyond the merely placebo effects, previous studies[24–27, 36–38, 52, 54]appear to be in line with our conclusions, supporting the idea that iCBT programs are effective in diminishing social anxiety, and modestly effective for comorbid anxiety and depression.Second, due to personnel constraints, post-intervention SCID interviews were not blinded regarding the study conditions. This could have influenced the study results in line with the interviewers’ expectations. However, similar results were obtained using social anxiety self-report measures at post-test. For example, similar recovery rates were obtained for SPIN (36.8%) and the full symptom remission on clinical interviews (34.2%). Furthermore, clinical significance change for the primary outcome measures yielded significant results, certifying that participants’ social anxiety decreased after the intervention. Third, the diagnostic procedure based on SCID interviews was conducted on telephone rather than in-person. However, we tried to overcome these limitations by using evidence-based screening tools with high psychometric properties (i.e., LSAS-SR, SPIN, BDI-II); and similar procedures were previously used in other clinical trials for selecting SAD participants [25, 36].Finally, the iSOFIE intervention was framed as an efficacy (e.g., how it works in well-controlled conditions), rather than as an effectiveness study (e.g., how it works in real clinical practice). However, this step is fundamental to support the internal validity of iCBT.

## Conclusions and Future Directions

These findings further support and replicate the efficacy of an iCBT program for SAD called *Internet Social Phobia (iSOFIE)*. The overall results of the iSOFIE group were superior compared to the WLCG, satisfaction with the treatment being high. Comparable ES on both primary and secondary outcome measures were obtained with both the long and the short version of the SOFIE treatment manual. Overall, it seems that the iSOFIE intervention can be effectively used to overcome the problem of high prevalence versus low access to evidence-based treatment for SAD (especially in developing countries).

To further advance the field of internet-delivered interventions future studies could include an active treatment condition, and investigate the external validity of such programs in psychiatric care. In this context, a tailored treatment might be needed in order to address potential co-morbid disorders [55]. A program such as the iSOFIE could be augmented with distinct modules created to respond to these needs. Also, a motivational interviewing could be added before the intervention to enhance treatment adherence [54, 56]. Nevertheless, as internet-delivered interventions display a high potential to widely disseminate effective evidence-based programs around the world, we conclude that iCBT for SAD works in Romania, and this novel treatment format holds promise as a treatment alternative in the future.

## Supporting Information

S1 CONSORT Checklist CONSORT Checklist. (DOCX)

S1 Dataset Multiple imputation. (SAV)

S2 Dataset Treatment satisfaction. (SAV)

S1 Protocol Protocol in English. (DOCX)

S2 Protocol Protocol in Romanian. (DOCX)
