# Supplementary material for: Internet-Delivered Cognitive-Behavioral Therapy for Social Anxiety Disorder in Romania: A Randomized Controlled Trial
Source: PLoS One. 2015 May 4;10(5):e0123997. doi: 10.1371/journal.pone.0123997 (PMC4418823; doi:10.1371/journal.pone.0123997)
Supplement: S1 Protocol — (DOCX) [file pone.0123997.s004.docx]

**The iSOFIE Research protocol**

**Background**

Anxiety represent one of the most common and debilitating conditions, affecting millions of lives around the world. The total annual cost for anxiety disorder has been estimated at $42 billion (Greenberg et al., 1999). In Western cultures, the lifetime prevalence of social anxiety disorders (SAD) ranges between 7-12% of the population (Furmark, 2002), with low rates of spontaneous remission (Keller, 2003), and prevalence appears to be increasing (Heimberg, Stein, Hiripi, & Kessler, 2000).

SAD often co-occurs with other psychiatric conditions such as mood disorders and substance use disorders, and is significantly associated with suicidal ideation (Eng & Heimberg, 2007; Olfson et al., 2000). For example, over 50% of people with social anxiety diagnosis also meet diagnostic criteria for other anxiety disorders and/or depression (Kessler et al., 2007).

There are a range of psychological and pharmacological treatments for SAD (Acarturk, Cuijpers, van Straten, & de Graaf, 2009). The most established psychological treatment for SAD is cognitive-behavioral therapy (CBT), which has proven effective (Heimberg, 2002). However, despite the availability of effective interventions, only a minority of individuals suffering from SAD seeks and receives appropriate treatment. Therefore, increasing the availability of evidence-based interventions for people suffering from SAD represents an important challenge (Andersson & Carlbring, 2011).

*Internet-delivered interventions*

Compared with face-to-face psychotherapies *internet-delivered interventions* present a unique set of inbuilt advantages that definitely recommend them for future use. These interventions lower many of the obstacles faced by ordinary patients, including finding a skilled psychotherapist, inconveniences of scheduling weekly face-to-face meetings, missing work or school hours, travel time and expenses, early access to treatment, inhibition reduction, embarrassment associated with help seeking and fear of what others might think or say and so forth. Internet-delivered interventions can also be provided at a low cost, with minimal effort needed from the health professionals. Therefore, internet-delivered interventions represent an efficacious, scalable, and cost-effective treatment option.

# Purpose and potential benefits of the study

The purpose of the current randomized controlled trial (RCT) is to implement an Internet-delevered cognitive behavioural therapy (iCBT) for people with social anxiety disorder. The intervention is based on the CBT principles adapted for the online environment. The present study aim is to test the efficacy of the *Internet Social Phobia* *program* (iSOFIE) in a culture where it was not tested before (i.e. Romania).

**Patient Selection and implementation**

The study will be advertised on the study web-page and in local newspapers. Potential participants will be invited read the informed consent, and if they agree, register for the study. An online screening procedure should be completed as the first step. The screening includ the following scales:

- Leibowitz Social Anxiety Scale – Self-Report version (LSAS-SR)
- The Social Phobia Inventory (SPIN)
- The Social Interaction and Anxiety Scale (SIAS)
- The Social Phobia Screening Questionnaire (SPSQ)
- Anxiety Sensitivity Index (ASI)
- Automatic Thoughts Questionnaire (ATQ)
- Attitude and Belief Scale-II (ABS-II)

Participants are expecred to be over 18 years old, free from psychosis and personality disorder, and not currently under psychological therapy. People who meet the mimimum inclusion criteria after the online screening will be contacted by phone for a short interview using the SCID.

After inclusion, participants will be randomized to eithe the ISOFIE group or to the wait list control group (WLCG). The active treatment (iSOFIE) is an intervention program with 9 modules presented in a previously determined order. The modules are akin to therapy sessions and homework assignments will be provided after every module. Participant’s activitie on the web-platform will be monitored by a psychologist who is supervised by a licensed psychotherapist or supervisor. After the iSOFIE group finalize 9-week intervention the WLCG participants will be invited to begin the program.

# Time schedule and evaluation

Preparations for the study will begein in the fall of 2011 (translation of the treatment manual, training of therapists etc.). A website will also be created and used during the study (Spring of 2012). Registered participants wil be screened and call for interview in late sping (April 2012). After randomizing participants to the experimental and control group, the 9-week treatment will start. Finally, a 6 months follow-up assessment will take place (Januarry 2013) and the collected data will be analized and interpreted.

**Project team**

Bogdan Tudor Tulbure, PhD, licensed psychologist, Department of Psychology, West University of Timișoara, Romania

Aurora Szentagotai, PhD, licensed psychologist, Department of Clinical Psychology and Psychotherapy, Babeş-Bolyai University, Romania

Oana David, PhD, licensed psychologist, Department of Clinical Psychology and Psychotherapy, Babeş-Bolyai University, Romania

Simona Ștefan, PhD, licensed psychologist, Department of Clinical Psychology and Psychotherapy, Babeş-Bolyai University

Daniel David, PhD, licensed psychologist, Department of Clinical Psychology and Psychotherapy, Babeş-Bolyai University, Romania

Kristoffer N. T. Månsson, PhD Student, Department of Behavioral Sciences and Learning, Linköping University, Sweden

Gerhard Andersson, PhD, licensed psychologist, Department of Behavioral Sciences and Learning, Linköping University, Sweden

A team of **11 master students** from Babeş-Bolyai University, Romania will conduct the screening and intervention under suprevision:

Bulea Alexandra, Calo Larisa Cristina, Golita Silvia, Isbășoiu Andreea, Pasca Flavia Sorana, Soflau Radu, Stan Alexandra, Stejar Carmina, Suta Darius, Teglas Rita Natasa, and Veresezan Emilia.

**References**

| Acarturk, C., Cuijpers, P., van Straten, A., & de Graaf, R. (2009). Psychological treatments of social anxiety disorder: a meta-analysis. *Psychological Medicine, 39,* 241-254. |
| --- |
| Andersson, G., & Carlbring, P. (2011). Social phobia (Social anxiety disorder). In C. Draper, & W. T. O’Donohue (Eds). *Stepped care and e-health: Practical applications to behavioral disorders* (pp. 99-114). New York: Springer. |
| Andersson, G., Carlbring, P., Holmström, A., Sparthan, E., Furmark, T., Nilsson-Ihrfelt E. …Ekselius, L. (2006). Internet-based self help with therapist feedback and in vivo group exposure for social phobia: a randomized controlled trial. *Journal of Consulting and Clinical Psychology, 74*(4)*,* 677-686. |
| Andersson, G., Carlbring, P., Holmström, A., Sparthan, E., Furmark, T., Nilsson-Ihrfelt, E., Buhrman, M., & Ekselius, L. (2006). Internet-based self-help with therapist feedback and in-vivo group exposure for social phobia: a randomized controlled trial. *Journal of Consulting and Clinical Psychology, 74*, 677-686. |
| Eng, W., & Heimberg, R. G. (2007). Social anxiety disorder. In C. Freeman & M. Power (Eds.), Handbook of evidence-based psychotherapies. A guide for research and practice (pp. 367-383). Chichester: John Wiley & Sons. Ltd. |
| Furmark, T. (2002). Social phobia: Overview of community surveys. *Acta Psychiatrica* *Scandinavica*, *105*(2), 84-93. |
| Greenberg, P. E., Sisitsky, T., Kessler, R. C., Finkelstein, S. N., Berndt, E. R., Davidson, J. R. T., et al. (1999). The economic burden of anxiety disorders in the 1990s. *Journal of Clinical Psychiatry, 60*, 427–435. |
| Heimberg, R. G. (2002). Cognitive-behavioral therapy for social anxiety disorder: current status and future directions. *Biological Psychiatry, 51*(1)*,* 101-108 |
| Heimberg, R. G., Stein, M. B., Hiripi, E., & Kessler, R. C. (2000). Trends in the prevalence of social phobia in the United States: A synthetic cohort analysis of changes over four decades. *European Psychiatry, 15*(1), 29-37 |
| Hofmann, S. G. & Otto M. W. (2008). *Cognitive Behavior Therapy for Social Anxiety Disorder: Evidence-based and Disorder Specific Treatment Techniques*, Taylor & Francis Group, NY. |
| Keller, M. B. (2003). The lifelong course of social anxiety disorder: A clinical perspective. *Acta Psychiatrica Scandinavica, 108 Supplement 417,* 85-94. |
| Kessler, R. C., Berglund P., Demler O., Jin, R., Koretz, D., Merikangas, K. R., Rush, A. J., Walters, E. E., & Wang, P. S. (2003). The epidemiology of major depressive disorder: results from the National Comorbidity Survey Replication (NCS-R). *Journal of the American Medical Association, 289,* 3095-3105 |
| Kessler, R. C., Merikangas, K. R., & Wang, P. S. (2007). Prevalence, comorbidity, and service utilization for mood disorders in the United States at the beginning of the twenty-first century. *Annual review of clinical psychology*, *3*, 137-158. |
| Olfson, M., Guardino, M., Struening, E., Schneier, F. R., Hellman, F., & Klein, F. (2000). Barriers to the treatment of social anxiety. *American Journal of Psychiatry, 157,* 521-527. |
