# Supplementary material for: Internet-Delivered Cognitive-Behavioral Therapy for Social Anxiety Disorder in Romania: A Randomized Controlled Trial
Source: PLoS One. 2015 May 4;10(5):e0123997. doi: 10.1371/journal.pone.0123997 (PMC4418823; doi:10.1371/journal.pone.0123997)
Supplement: S2 Protocol — (DOCX) [file pone.0123997.s005.docx]

Universitatea Babeş-Bolyai

Facultatea de Psihologie şi Ştiinţele Educaţiei

**Către Board-ul de Recenzie Instituţională**

**pentru acordarea vizei etice**

**Protocolul studiului**

**a. Titlul proiectului:**

Terapia cognitiv comportamentală a fobiei sociale în mediul virtual: Un studiu clinic controlat^[[1]](#footnote-1)^

**b. Rezumatul proiectului:**

Înainte de a oferi orice intervenţie psihologică, este important să testăm eficaţitatea ei într-un studiu clinic controlat. Scopul acestei investigaţii este de-a testa eficienţa unei interveţii pe internet destinată reducerii nivelului de anxietate socială. În cadrul acestei intervenţii participanţii (i.e., persoane diagnosticate cu anxietate socială conform criteriilor DSM-IV) au ocazia de-a îşi înţelege mai bine condiţia, de-a identifica şi restructura gândurile automate negative, dar şi de a implementa o serie de exerciţii de expunere care s-au dovedit foarte eficiente în reducerea simptomatologiei anxioase. Cele nouă module care vor fi prezentate online au fost elaborate pe baza principiilor Terapiei Cognitiv-Comportamentale (TCC). Programul este compus dintr-un protocol de evaluare şi un protocol de interveţie. Studii similare efectuate în Suedia şi Elveţia au demonstrat eficacitatea acestor intervenţii.

**c. Implicaţii etice pentru subiecţi umani:**

Participarea la acest studiu este voluntară şi nu pune în nici un fel în pericol siguranţa subiecţilor. Participanţii vor fi încurajaţi să raporteze persoanei de contact orice dificultăţi întâmpinate pe parcursul studiului. Sugestiile vor fi oferite fie telefonic, fie printr-un mesaj scris (vezi subcapitolul privind confidenţialitatea datelor). Participanţii au dreptul în orice moment să se retragă din studiul dacă doresc acest lucru. Designul studiului face ca riscurile de durere şi disconfort fizic să fie foarte scăzute. Cu toate acestea, orice intervenţie psihologică presupune anumite schimbări ale obiceiurilor de viaţă. Aceste informaţii sunt oferite chiar de la început, înainte de începerea intervenţiei propriuzise. În plus, dacă participanţii sunt preocupaţi de securitatea datelor, vor putea citi despre felul în care sunt înregistrate şi stocate datele cu caracter personal pe site-ului proiectului, înainte de a decide să participe.

O preocupare constantă a celor care oferă servicii psihologice este managementul riscului suicidar. Participanţii care în faza de screening prezintă risc suicidar vor fi direcţionaţi spre alte tratamente, de preferinţă psihiatrice. În cazul în care este detectată prezenţa riscului suicidar, participanţii sunt ajutaţi să contacteze serviciile corespunzătoare de sănătate mintală. De asemenea, pe toată desfăşurarea studiului, participanţii care au nevoie urgentă de ajutor sunt încurajaţi să ia legătura telefonic sau prin e-mail cu terapeuţii implicaţi în studiu pentru a lua măsurile adecvate.

Persoanele care vor fi excluse din studiu ar putea percepe acest lucru ca un fapt negativ. Pentru a preveni astfel de reacţii, o descriere clară a criteriilor de includere va fi afişată pe site-ul proiectului. Persoanele excluse datorită altor condiţii psihologice/medicale care nu sunt tratate prin intermediul acestui program vor fi îndrumate să consulte alte servicii psihologice sau medicale.

Alte riscuri posibile: Este posibil ca unii participanţi să nu beneficieze de această intervenţie în cuida timpului şi efortului investit. Cu toate acestea, noi considerăm că acest risc este semnificativ mai mic decât şansa de-a obţine o reducere substanţială a simptomatologiei. În plus, riscul efectelor nule nu este mai mare în cadrul intervenţiilor online decât în cadrul intervenţiilor psihoterapeutice clasice.

Confidenţialitatea datelor: Fiecărui participant i se va atribui un număr care va fi utilizat de ficare dată când se va comunica cu acesta pe Internet. Corespondenţa va fi mediată prin intermediul unui software securizat care funcţionează pe aceleaşi principii ca Internet banking-ul. Toţi participanţii vor primi un cod personal care le va permite accesul în sistem. Adresele personale de email nu vor fi folosite pentru a comunica direct cu participanţii (i.e., comunicarea se va face exclusiv în interiorul sistemului). Un software encriptat va facilita corespondenţa dintre participanţi şi terapeuţi, iar codul personal şi codurile unice vor asigura folosirea exclusivă a sistemului doar de persoanele autorizate. Datele personale ale participanţilor şi cheia de encriptare, care face legătura între ID-ul lor şi studiu, vor fi salvate pe un stick USB care este păstrat într-un dulap încuiat la Linkoping University, Suedia. Pentru a asigura confidenţialitatea datelor, informaţiile cu caracter personal de pe stick-ul USB vor fi encriptate şi protejate cu o parolă. De asemenea, datele din chestionare vor fi înregistrate în mod anonim, ceea ce înseamnă că participanţii nu vor putea fi identificaţi după efectuarea fazei de screening.

**d. Metodologia:**

**i. Obiectivul propiectului:** Principalul obiectiv al acestui studiu este de-a testa în ce măsură administrarea unei intervenţii online bazată pe principiile TCC va avea efecte statistice şi clinice semnificative în cazul adulţilor diagnosticaţi cu fobie socială.

**ii. Subiecţii utilizaţi:** Participanţii vor fi recrutaţi prin intermediul unor anunţuri media (i.e., pe internet, în ziare locale şi naţionale, pe o pagina de Facebook deschisă special pentru acest scop etc.). Persoanele interesate vor putea accesa pagina de web a proiectului, unde vor răspunde la chestionarele de screening. Vor fi incluse doar persoanele care în urma screening-ului şi a interviului telefonic vor satisface concomitent următoarele criterii: au peste 18 ani, au acces la internet, sunt vorbitoare de limbă română, nu prezintă ideaţie suicidară în urma chestionarului BDI şi a interviului clinic, satisfac criteriile DSM-IV pentru anxietatea socială (în urma interviului clinic). Vor fi excluse persoanele care abuzează de substanţe şi / sau au tulburări psihice severe. Un alt criterii de excludere reprezintă folosirea medicamentelor psihotrope dacă doza nu a fost constantă timp de cel puţin o lună^[[2]](#footnote-2)^. Pornind de la mărimea efectului constatată în cercetări similare (*d* = 0,70) estimăm că în acest studiu este nevoie de 80 de participanţi (i.e., 40 pentru fiecare condiţie experimentală). Toate persoanele care *nu* vor fi incluse în studiu vor primi un mesaj personalizat, împreună cu informaţii suplimentare despre alte locuri unde ar putea solicita ajutor.

**iii. Materiale/Instrumente:** Pentru selecţia participanţilor şi evaluarea eficaţităţii intervenţie vor fi folosite următoarele instrumente din litaratura de specialitate: Inventarul Fobie Sociale (engl. Social Phobia Inventory SPIN), Scala de Anxietate Socială Leibowitz (engl. Leibowitz Social Anxiety Scale LSAS), Scala de Interacţiuni şi Anxietate Socială (engl. Social Interaction and Anxiety Scale SIAS), Chestionarul de Screening pentru Fobia Socială (engl. Social Phobia Screening Questionnaire SPSQ), Interviul Clinic Structurat (Structured Clinical Interview SCID for DSM-IV), Inventarul de Depresie Beck (Beck Depression Inventory BDI), Scala de Atitudini şi Convingeri II (Attitude and Belief Scale II, ABS II). Toate instrumentele au proprietăţi psihometrice adecvate, şi au fost utilizate în studii similare.

**iv. Procedura:**

Persoanele care doresc să participe la acest studiu vor fi informate despre intervenţie prin intermediul paginii de web a proiectului. Înainte de începerea prorpriu-zisă a studiului, participanţii vor fi îndrumaţi să citească Consimţământul Informat, să îşi exprime acordul de-a participa. Abia apoi vor putea să completeze online chestionarele de screening.

Odată incluşi în studiu, participanţii vor fi randomizati în una din cele două condiţii experimentale: a) iSOFIE sau b) grupul de control. Programul iSOFIE constă din nouă module prezentate online. Module iSOFIE au fost elaborate respectând principiile TCC pentru fobia socială. Fiecare modul este sctucturat asemenea unei şedinţe de terapie, şi conţine cel puţin o temă de casă. Participanţii din ambele grupe vor completa săptămânal scala LSAS. Fiecărui participant îi va fi alocat un terapeut online, care este fie un masterand în domeniul psihologie care lucrează sub supervizare, fie un psiholog autorizat (i.e., autonom, specialist sau principal).

După completarea post-testului la sfârţitul celor nouă săptămâni, participanţii din grupul de control vor avea posibilitatea de-a parcurge modulele iSOFIE. Toţi participanţii vor fi contactaţi la 6 luni după terminarea tratamentului, şi rugaţi să completeze toate chestionarele incluse în studiu (i.e., SPIN, LSAS, SIAS, SPSQ, SCID, BDI, şi ABS II).

**v. Rezultate scontate:** Participanţii vor avea posibilitatea să parcurgă online principalele etape ale TCC pentru fobia socială, într-o modalitate asemănătoare unei intervenţii psihologice convenţionale. Ne aşteptăm ca majoritatea participanţilor să obţină o reducere a simptomatologiei, deoarece metodele utilizate sunt cunoscute în literatură ca fiind eficiente. Sub aspect ştiinţific, rezultatele obţinute vor fi trimise spre publicare în reviste naţionale şi internaţionale de prestigiu. Pe baza datelor culese, ne aşteptăm să trimitem spre publicare cel puţin un articol ştiinţific către o revistă cotată ISI.

Postdoctorand: Bogdan Tudor Tulbure Mentor: Conf. dr. Aurora Szentagotai

Coordonator program: Prof. dr. Daniel David

Coordonator Linkoping University: Prof. dr. Gerhard Andersson ([www.gerhardandersson.se](http://www.gerhardandersson.se))

**Notă:** Acest studiu este finanţat prin proiectul **POSDRU 89/1.5/S/60189** cu titlul „Postdoctoral Programs for Sustainable Development in a Knowledge Based Society”.

1. Acest studiu reprezintă rezultatul cooperării între Universitatea Babes-Bolyai şi Lynkoping Univeristy, Suedia. Platforma online pe care o vom folosi a fost utilzată cu succes în peste 15 studii clinice controlate efectuate în Suedia, Germania, şi Africa de Sud. [↑](#footnote-ref-1)
2. Pe parcursul studiului participanţii au dreptul să consulte medicul psihiatru, şi să efectueze modificări ale tratamentului medicamentos sub întrumarea acestuia. Solicitarea noastră este ca, dacă acest lucru se întâmplă, participanţii să informeze în timp util echipa de cercetare. Participanţii care şi-au modificat tratamentul medicamentos pe parcursul studiului vor beneficia în continuare de tratamentul online, însă datele lor vor fi excluse din analizele statistice. [↑](#footnote-ref-2)
